# 3,4-dihydroxyphenylethyl alcohol glycoside reduces acetaminophen-induced acute liver failure in mice by inhibiting hepatocyte ferroptosis and pyroptosis

**DOI:** 10.7717/peerj.13082

**Published:** 2022-03-14

**Authors:** Tianyu Liu, Lei Yang, Hejun Gao, Yuzhen Zhuo, Zhengwei Tu, Yongqin Wang, Jing Xun, Qi Zhang, Lanqiu Zhang, Ximo Wang

**Affiliations:** 1 Tianjin Medical University, Tianjin, China; 2 Tianjin Nankai Hospital, Tianjin, China; 3 The Affiliated Hospital of Guilin Medical University, Guilin, Guangxi, China; 4 Integrated Chinese and Western Medicine Hospital Of Tianjin University, Tianjin, China

**Keywords:** Acetaminophen, Acute liver failure, Ferroptosis, Pyroptosis, 3,4-dihydroxyphenylethyl alcohol glycoside

## Abstract

APAP is one of the most commonly used antipyretic and pain medications, but excessive use can cause liver toxicity and damage. 3,4-dihydroxyphenylethyl alcohol glycoside (DAG) is a component isolated from *Sargentodoxa cuneata* known to have anti-apoptotic, anti-oxidation and anti-inflammatory effects. However, the effects of DAG on acute liver failure (ALF) are largely unknown. The purpose of this study is to study the protective effects and mechanism of DAG on APAP-induced ALF in mice. We established an ALF model in adult male pathogen-free C57BL/6 mice treated with APAP (300 mg/kg) by intraperitoneal injection and resolved by 24 h. Hematoxylin and eosin (HE) staining was used to evaluate the pathological changes in mouse liver tissue. The infiltration of neutrophils in liver tissue and reactive oxygen species (ROS) in AML12 cells were analyzed by flow cytometry. The levels of alanine aminotransferase (ALT), aspartate aminotransferase (AST), glutathione (GSH), malondialdehyde (MDA), catalase (CAT), and superoxide dismutase (SOD) were analyzed using relevant kits. Our results show that DAG reduced APAP-induced serum ALT and AST levels, histopathological changes, liver neutrophil infiltration and proinflammatory cytokines production, also attenuated the accumulation of MDA and the exhaustion of GSH, CAT and SOD. *In vitro* experiment indicated that DAG dose-dependently inhibited APAP-induced the levels of pro-inflammatory factors (IL-1β and IL18), and reactive oxygen species (ROS) and preventing GSH depletion in mouse AML12 hepatocytes. More interestingly, DAG inhibited the expression of ERK, HO-1, NLRP3, Caspase1 (p20) and Gasdermin-D and upregulated the expression of GPX4 in liver tissues and AML12hepatocytes. Therefore, our results indicate that DAG may act as a potential agent to treat ALF induced by APAP by inhibiting hepatocyte ferroptosis and pyroptosis.

## Introduction

The liver is the largest physical organ in the body. It is essential for glycogen storage, drug detoxification, metabolic control, cholesterol synthesis and transport regulation, urea metabolism, and the secretion of various plasma proteins (Si-Tayeb, Lemaigre & Duncan, 2010). Drug-induced liver disease is very complex, and in most cases it is not clear. Some drugs have direct toxic effects, liver damage caused by them is generally predictable, and the damage is related to the dose of the drug and is unique to some drugs (Jiménez Sánchez, Serrano Díaz & Martínez Crespo, 2020).

Acetaminophen (APAP) is one of the commonly used antipyretic and analgesic drugs in clinical practice. However, when APAP is overdose, the risk of liver toxicity and acute liver failure (ALF) also increases. In Western countries, the leading cause of ALF is still APAP overdose (Stravitz & Lee, 2019). The core mechanism of ALF caused by APAP is mainly oxidative stress and mitochondrial dysfunction (Iorga & Dara, 2019). N-acetylcysteine (NAC) is the only drug recommended by the US Food and Drug Administration for the treatment of patients with APAP overdose. Still, it has limitations due to its narrow therapeutic window and side effects (Du, Ramachandran & Jaeschke, 2016). It is this limitation that is likely to lead to missing the appropriate treatment stage, and then the only option to improve the survival rate of ALF patients becomes liver transplantation (Craig et al., 2010). It has been found that the liver toxicity caused by APAP mainly includes oxidative stress, aseptic inflammation and many other aspects. Many genes or molecules have been determined to play a vital role in this process, and they can be used as potential targets for the treatment of APAP-induced hepatotoxicity. In recent years, studies have shown that natural products have a protective effect on APAP-induced liver toxicity, providing many alternative drugs for the treatment of ALF (Yan et al., 2018).

In many traditional Chinese medicines, including *Sargentodoxa cuneata*, phenethyl steroid glycoside (PhG) 3, 4-dihydroxy phenethyl alcohol glycoside (DAG) has been identified. *Sargentodoxa cuneata* is a famous traditional Chinese medicine (TCM) used to treat rheumatoid arthritis, ulcers, acute appendicitis, amenorrhea and menstrual pain (Zhang et al., 2017). PhG is a kind of water-soluble compound, which is characterized by cinnamic acid and hydroxyphenylethyl groups connected with β-glucopyranose /β-fructopyranose through ester bond and glycoside bond respectively (Xue & Yang, 2016). PhG has a variety of pharmacological properties, including anti-inflammatory and anti-apoptotic effects (Li et al., 2018a; Li et al., 2018b), antioxidant activity (Harput, Genc & Saracoglu, 2012). Previously, we have shown that DAG is one of the active ingredients Staphylococcus kluyveri with high-performance liquid chromatography (HPLC) (Li et al., 2016). Our studies have demonstrated that DAG isolated from *S. cuneata* has a variety of beneficial effects. DAG can be used as anti-inflammatory drugs, such as against colitis (Zhuo et al., 2019) and acute lung injury (Li et al., 2019). So, we speculate that DAG can reduce acetaminophen-induced ALF.

## Materials and Methods

### Animals

Fifteen C57BL/6 mice were purchased from Beijing Huafukang Biotechnology Co., Ltd (Beijing, China). Mice are 6–8 weeks old and weigh about 25–30 g. The mice were placed in the breeding cages of the experimental animal room, and the mice were allowed to eat and drink freely. After enabling the mice to adapt to the environment for a week, they were ready to experiment. This research protocol has been approved by the Medical Ethics Committee of Tianjin Nankai Hospital and complies with all animal health and handling guidelines issued by the committee (NKYY-DWLL-2021-050).

### Drug and reagents

DAG was previously isolated from *S.cuneata* by our laboratory  (Li et al., 2019). HPLC showed that the purity of the separated DAG was >97%. APAP was purchased from (Aladdin, Shanghai, China). APC anti-CD45, FITC anti-CD11b and PE anti-Ly6G were purchased from Invitrogen (Elabscience, Wuhan, China) for flow cytometry experiments. For detecting the concentration of liver tissue protein, BCA protein assay kit was purchased from (Thermo Scientific, Waltham, MA, USA).

### Animal treatments APAP

The 6–8 weeks old C57BL/6 mice were randomly divided into three groups (five in each group). The three groups were control group, model group (APAP; 300 mg/kg), and treatment group (APAP+DAG; APAP 300 mg/kg; DAG 100 mg/kg). The model group was intraperitoneally injected with 100ul normal saline before 200 ul APAP. The treatment group was intraperitoneally injected with 100ul DAG before 200 ul APAP. Mice were anaesthetized after treatment with chloral hydrate, liver tissue and blood samples were collected for subsequent analysis after 24 h following APAP treatment.

### Cell experiment

Murine hepatocyte AML12 cells (HuZhen Biotechnology Co., Ltd, Shanghai, China) were cultured in DMEM/F12 medium supplemented with 10% fetal bovine serum and maintained at 37 °C with 5% CO2. The cells were pretreated with the different doses of DAG (50, 100, 150 µM, respectively) 30 min before APAP (10 µM) administration for 24 h, followed by analysis for the subsequent step study.

### Histopathological examination

After obtaining mouse liver tissue as described above, the left lobe of the liver was taken and fixed with 10% formalin. Then, embedded in paraffin for HE staining section preparation (all animals were not dead, and the materials were taken under animal anesthesia). Then the morphological examinations of the liver were observed under an optical microscope and saved by photographs.

### Plasma ALT and AST activity assays

ALT and AST activity assay kits (Jiancheng, Nanjing, China) were used to detect the activity changes of ALT and AST in mouse serum. After adding mouse serum and reagents to the 96-well plate, placed at room temperature for 15 min, and measured the OD value at a wavelength of 510 nm with a microplate reader.

### Flow cytometry analysis

Under the anesthesia of the mice, we took part of the right lobe of the liver, digested with 0.05 mg/mL type IV collagenase, hyaluronidase and DNase I at 37 °C for 30 min. The fragments of liver tissue were ground through a 70 µm filter in 10 ml PBS, we took precipitation after centrifugation, and resuspended the cells in 5 ml PBS. After removing the red blood cells with 5ml red blood cell lysis buffer, the hepatocytes were stained (anti-CD45-APC, anti-CD11b-FITC and anti-Ly6G-PE) for 30 min (4 °C) in the dark. Next, the cells were washed in PBS (1 mL) and then discarded the supernatant, added 200 µl PBS and shook. AML12 cells were digested for 5 min, stained with DCFH-DA (10 µM) (Solarbio Biotech Co., Beijing, China) for 20 min, and then washed twice with PBS. NovoCyte flow cytometer (Dakewe Biotech Co., Shenzhen, China) was used for detection, and analyzed the results with Flowdroid software. Neutrophils were marked as CD45 + CD11b + Ly6G +.

### Hepatic lipid peroxidation and antioxidative activity assay

In order to study the lipid peroxidation and antioxidant effect of DAG in mice, a commercial kit (Solarbio, Beijing, China) was used to measure the activity of antioxidants in the liver, including Catalase and SOD. After adding tissue homogenate and reagents to the 96-well plate, they were placed at room temperature for 30 min, and measured the OD value at a wavelength of 240 nm and 560 nm with a microplate reader.

### Quantitative real-time PCR (qPCR)

Following the manufacturer’s instructions to extract total RNA from liver tissue and AML12 cells using Trizol reagent (Takara, Japan). The purity of the RNA was verified by spectrometer (Thermo Fisher Scientific, MA, USA) at 260 nm. Then TransGen reverse transcription was used to reverse transcript from RNA (1 µg each) to cDNA for qPCR (Applied Biosystems). SYBR Mix (Yeasen, Shanghai, China) were used to measure the mRNA expression levels of IL-1β, IL-18, and NLRP3 by qPCR. The relative expression level of mRNA was normalized to GAPDH. The primer sequence is as follows: IL-18 (forward primer: 5′-AGTTGCCTTCTTGGGACTGA-3′; backward primer: 5′-TCCACGATTTC CCAGAGAAC-3′), IL-1β (forward primer: 5′-CTATGTCTTGCCCGTGGAG-3′; backward primer: 5′-CATCATCCCACGAGTCACA-3′), NLRP3 (forward primer: 5′-CGTCAGCCGATTTGCTATCT-3′; backward primer: 5′-CGGACTCCGCAAAGTCTAAG-3′), GAPDH (forward primer: 5′- GCCTCGTCTCATAGACAAGATG-3′; backward primer: 5′- CAGTAGACTCCACGACATAC -3′).

### Glutathione assay

Mice liver tissue or AML12 cells were homogenized. The supernatants were obtained to measure the OD value according to the instructions of the reagent manufacturer (Jiancheng Biotech Co., Nanjing, China). The relative contents of glutathione (GSH) were used for follow-up analysis.

### Western blot analysis

Part of the right lobe of the mouse liver tissue was weighed and cut into pieces. The mouse liver tissue sample or AML12 cells were lysed on ice in RIPA buffer (Roche Diagnostics, Basel, Switzerland) containing a mixture of phosphatase inhibitor and serine protease inhibitor for 30 min. After that, the lysate was centrifuged at 12,000 g for 15 min at 4 °C. The sediment was discarded, and the supernatant was taken. The BCA protein assay kit (Thermo Fisher Scientific, Waltham, MA, USA) was used to determine the protein concentration in the supernatant. The protein was denatured and separated by a 10% SDS-PAGE gel, and then transferred to a PVDF membrane (Miripoli, Massachusetts, USA). At room temperature, the membrane was sealed with 5% (w/v) skimmed milk for 2 h. Next, the membrane was incubated with the primary antibody overnight at 4 °C: ERK, p-ERK, HO-1, GPX4, NLRP3, Gasdermin-D (Cell Signaling Technology, MA, USA), Caspase 1 (p20) (Santa Cruz, CA, USA), which would combine HRP. The membrane was incubated with the secondary antibody for 2 h, the membrane was washed five times with TBST, and the protein was visualized using an enhanced chemiluminescence method (Yeasen, Shanghai, China). Imager (Tanon, Shanghai, China) was used to image and quantify the blot.

### Statistical analysis

Analysis of variance was used to compare the differences between the groups. The data were expressed as mean ± standard deviation (SD). Use GraphPad Prism 8 (GraphPad Software, USA) to perform a one-way analysis of variance. All statistical analysis of the above data uses *t*-test. We believed that *p*-value <0.05 was considered statistically significant.

## Result

### Effects of DAG on relative liver weight and liver histopathological changes in mice with acetaminophen-induced ALF

**Figure 1:**
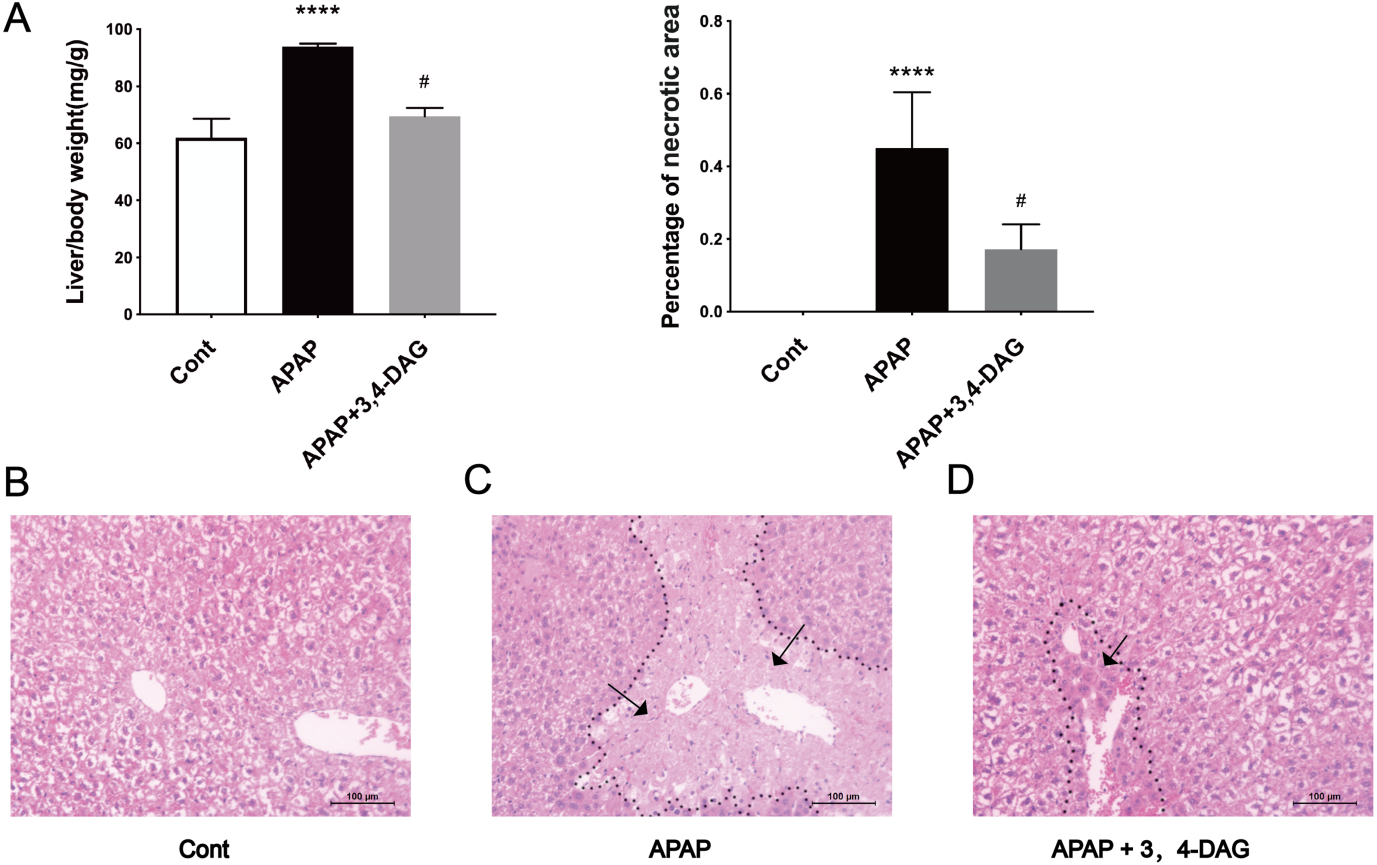
Effects of DAG on relative organ weight and liver histopathological (A) Effects of DAG on relative organ weight (*n* = 5 per group, *df* = 4, ****p < 0.0001, ^#^*p* < 0.0001). APAP compared with control; DAG compared with APAP. (B–D) Liver tissue sections stained with hematoxylin.

The mouse model of APAP-induced ALF was used to explore the potential effects of DAG. As shown in Fig. 1A, compared with the APAP group, a significant decrease in the relative weight of the liver was observed in the DAG + APAP group after 24 h treatments. Compared with the control group, the relative liver weight of APAP injection increased significantly (*p* < 0.01). In addition, we evaluated the liver sections stained with haematoxylin and eosin (HE). The liver of the mice in the control group had no pathological abnormalities (Fig. 1B). In contrast, APAP treatment resulted in some histopathological changes in the liver, such as destroyed hepatic lobule, significant cell necrosis, loss of hepatocyte structure around blood vessels, and lymphocyte infiltration (Fig. 1C). However, pre-administration of DAG can improve liver necrosis and the relative intactness of hepatic lobule structure was maintained (Fig. 1D). (The necrotic area has been circled with black curve and has been made statistics).

### Effect of DAG on liver function

**Figure 2:**
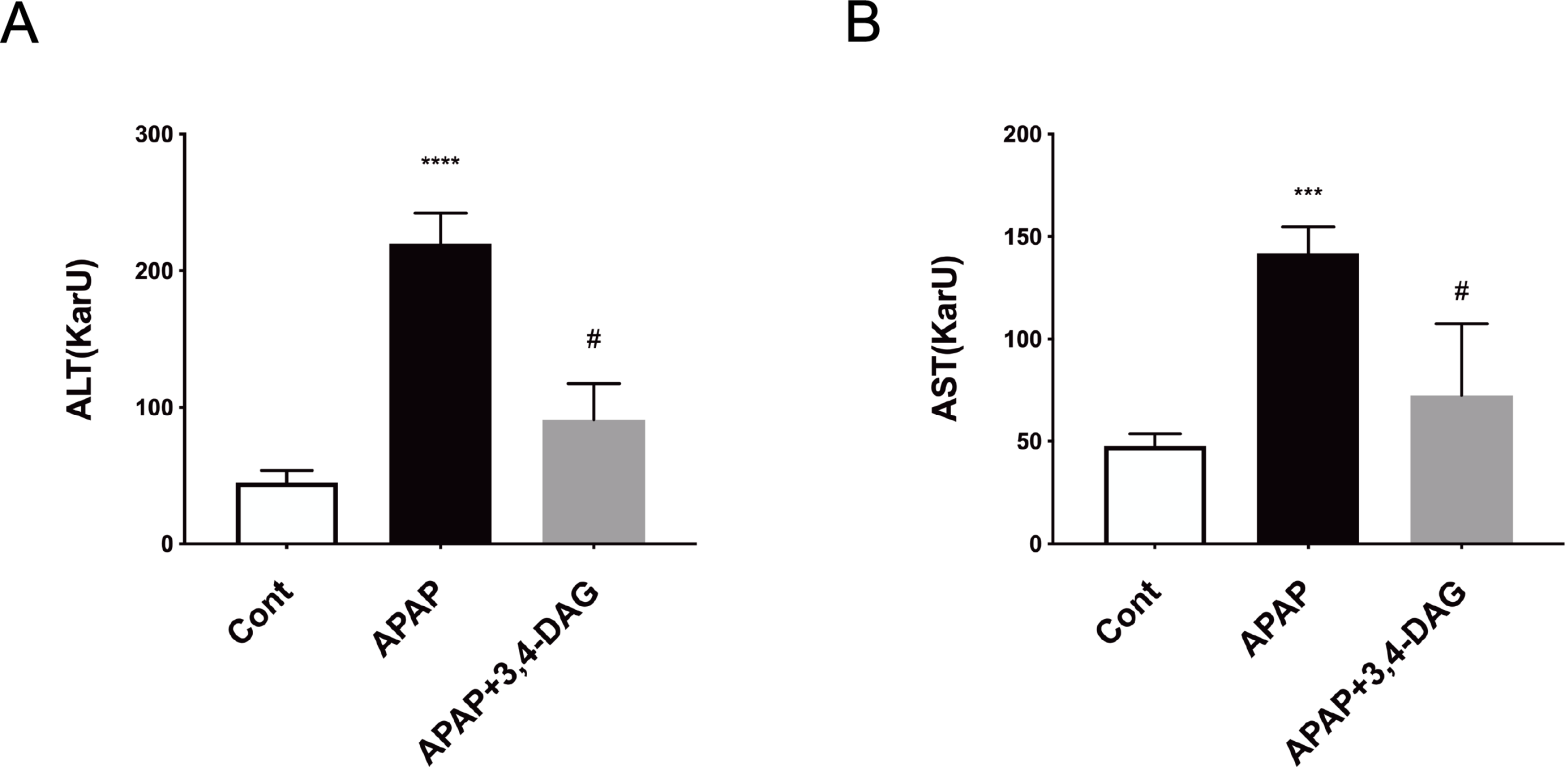
Effect of DAG on activities of plasma ALT (A) and AST (B) in APAP-treated mice. ****p < 0.0001 APAP group compared with control. ^#^*p* < 0.0001 DAG compared with APAP group. (*n* = 5 per group, *df* = 4).

As shown in Fig. 2, compared with the control group, the levels of ALT and AST in plasma was significantly increased after injection of APAP. In contrast, DAG treatment significantly suppressed the APAP-induced increase in the activities of ALT and AST to improve liver function (*p* < 0.01).

### Effect of DAG on hepatic antioxidative activity on mice

**Figure 3:**
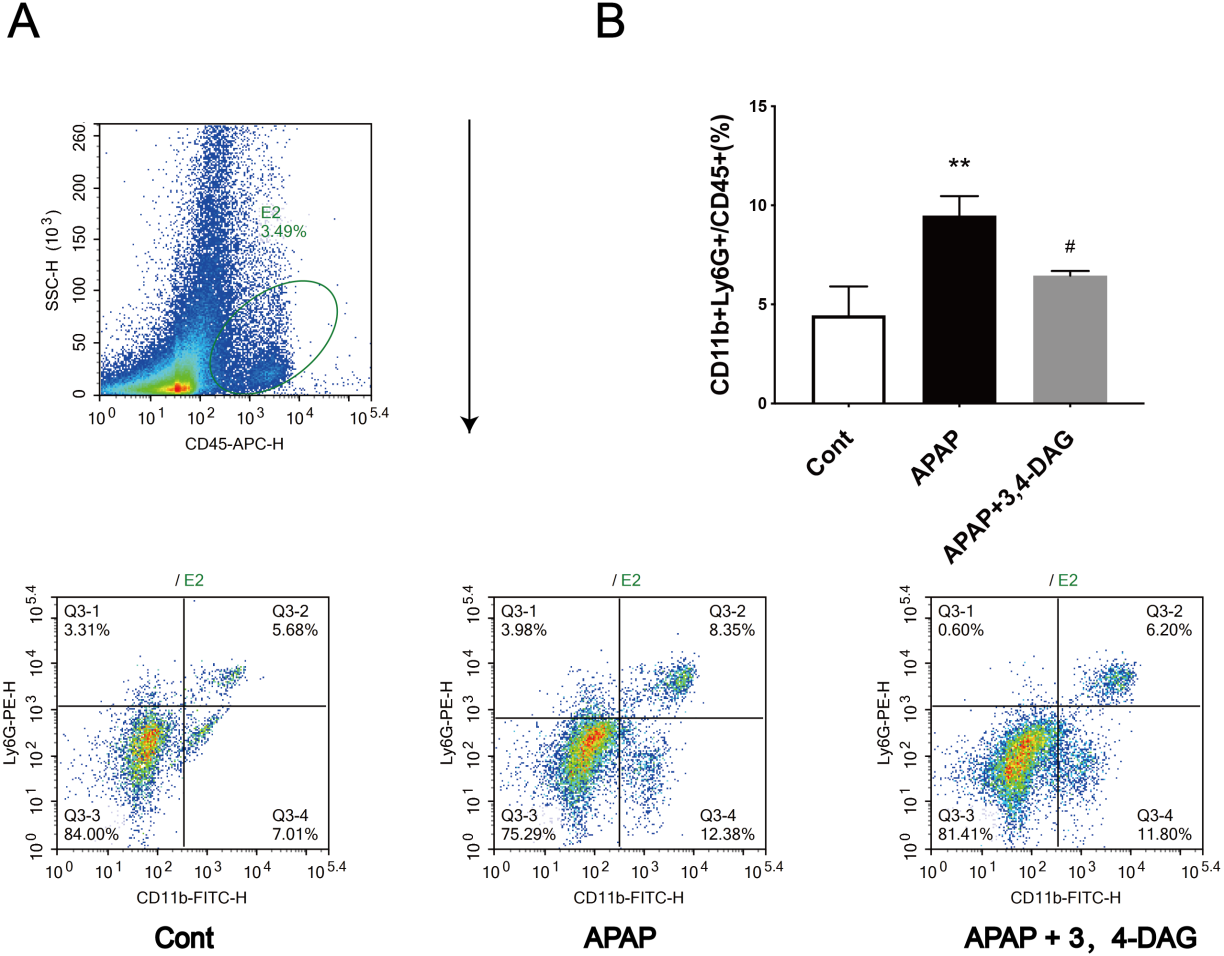
Effect of DAG on APAP-induced hepatotoxicity with neutrophil aggregation in mice. Cells from Livers were harvested and analyzed using flow cytometry 24 h after hematoxylin. The percentage of neutrophil in the liver (A). Data presented are the mean ± SD of the percentage of neutrophil in the liver (B). **p = 0.0086, APAP compared with control; ^#^*p* = 0.0258, DAG compared with APAP. (*n* = 3, *df* = 2).

It has been demonstrated that enhanced oxygen free radicals play critical roles in mediating the hepatotoxicity induced by APAP (Mason & Fischer, 1986). Thus, we firstly examined the effect of DAG on SOD, catalase activity and GSH. As a result, APAP treatment significantly reduced the activity of SOD and catalase. In contrast, pretreatment with DAG significantly inhibited the decrease in the activity of SOD and catalase induced by APAP. Compared with the control group, SOD and catalase were lower in the APAP group (Fig. 3, *p* < 0.05). On the contrary, treatment with 100 mg/kg/day DAG compared with the APAP group, SOD and catalase were significantly increased. (Fig. 3, *p* < 0.01). The content of GSH also showed similar results. (Fig. 3, *p* < 0.01). These results indicated that DAG could exert its antioxidant effect to protect APAP-induced hepatotoxicity in mice.

### Effect of DAG on APAP-induced ferroptosis

**Figure 4:**
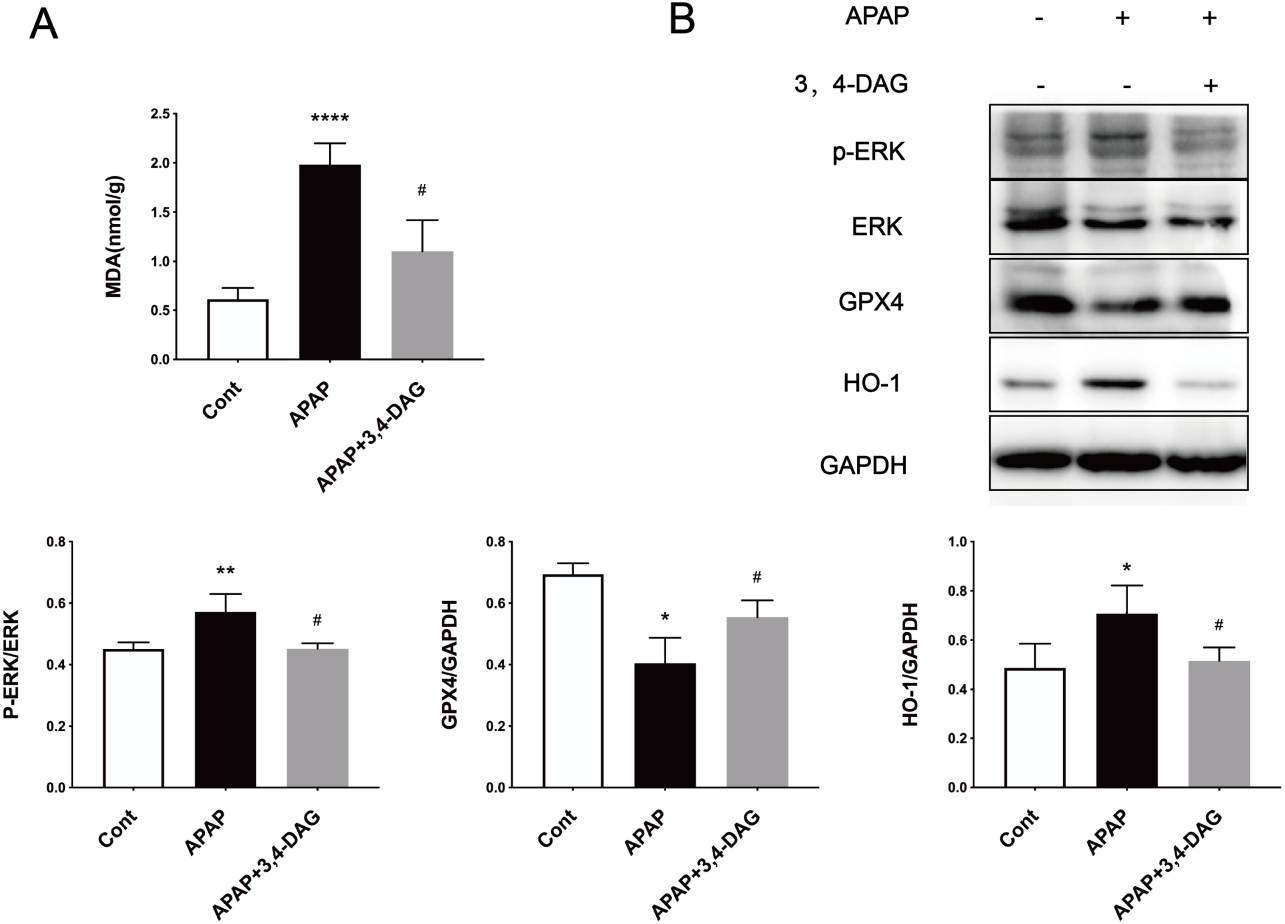
Effects of DAG on lipid per oxidation in the liver of APAP-treated mice and treatment on ERK, GPX4 and OH-1 activation in the liver tissues of mice with APAP-induced hepatotoxicity (*n* = 4 per group, *df* = 3). MDA levels of DAG are decreased compared with APAP. (**p < 0.0001 APAP compared with control, ^#^*p* = 0.0012 compared with DAG). Proteins extracted from the liver were identified using western blot. DAG decreased the levels of p-ERK and HO-1, whereas it increased the levels of GPX4 (B).

Ferroptosis is a regulated form of cell death that relies on iron and reactive ROS and is characterized by lipid peroxidation (Latunde-Dada, 2017). Recent studies have shown that ferroptosis plays an essential role in the pathogenesis of acute liver failure (Yamada et al., 2020). As shown in Fig. 4A, compared with the control group, the MDA level is significantly increased in the liver of the APAP group (*p* < 0.0001). In the group treated with DAG, it was observed that APAP injection significantly reduced the MDA content (*p* < 0.01). In order to determine whether anti-lipid peroxidation was involved in the suppressive effect of DAG on APAP-induced ALF, we analyzed the ERK, GPX4, and HO-1 signaling pathways by western blot. We confirm that the APAP treatment significantly decreased the GPX4 expression in the liver tissue. However, pretreatment with DAG significantly suppressed this inhibition induced by APAP. The APAP treatment significantly increased the HO-1 and GPX4 expression in the liver tissue. However, pretreatment with DAG significantly reversed the above trend (Fig. 4B). Studies have shown that HO-1 and GPX4 are involved in the process of ferroptosis. Therefore, these results indicate that DAG may inhibit APAP-induced lipid peroxidation on mouse liver tissues by alleviating ferroptosis.

### Effects of DAG on APAP-induced neutrophil infiltration

**Figure 5:**
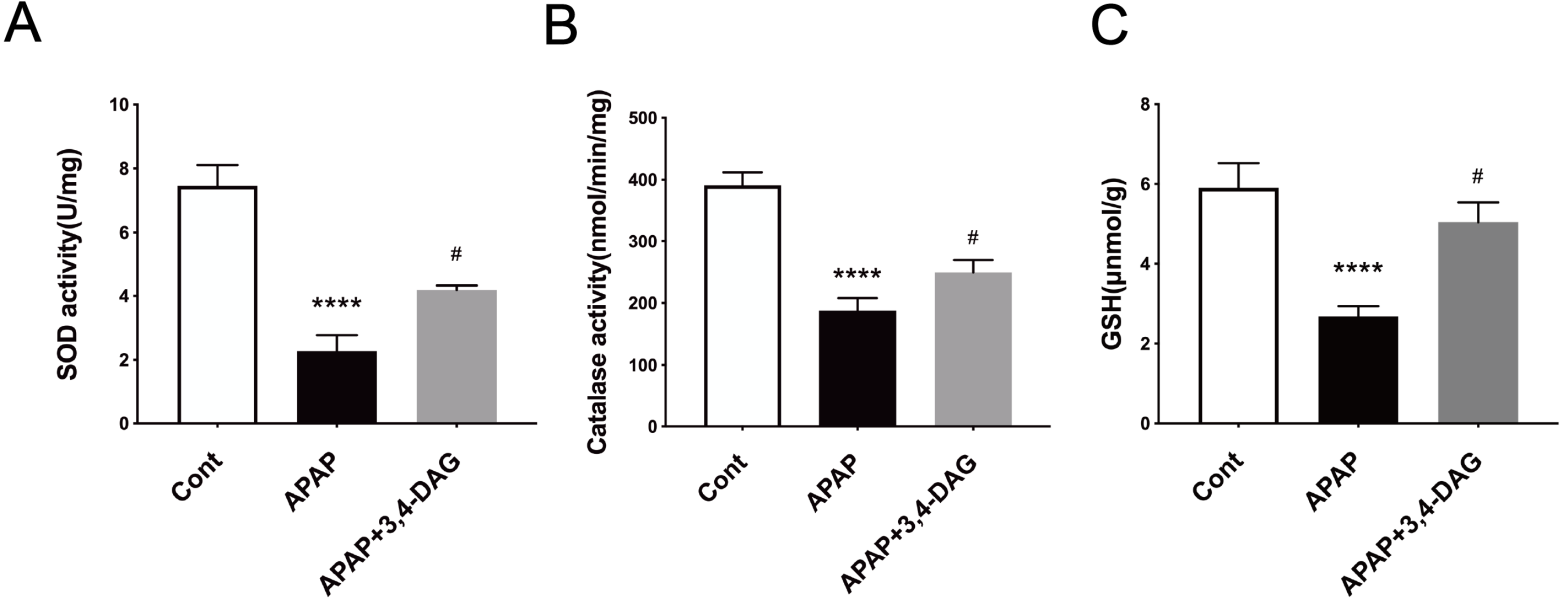
Effects of DAG on the activities of SOD (A), catalase (B) in the liver of APAP-treated mice (*n* = 4 per group, *df* = 3). ****p < 0.0001 indicate a statistically significant difference when APAP group compared with control, and ^#^*p* = 0.0009 (A)/^#^*p* = 0.0054 (B) compared with DAG.

Flow cytometry was used to analyze the neutrophil infiltration in the live tissue. The results showed that APAP alone led to a significant increase in the percentage of neutrophils in the liver compared with the control group. However, DAG treatment significantly reduced the increased percentage of neutrophils induced by APAP (Fig. 5).

### Effect of DAG on APAP-induced hepatocyte pyroptosis

**Figure 6:**
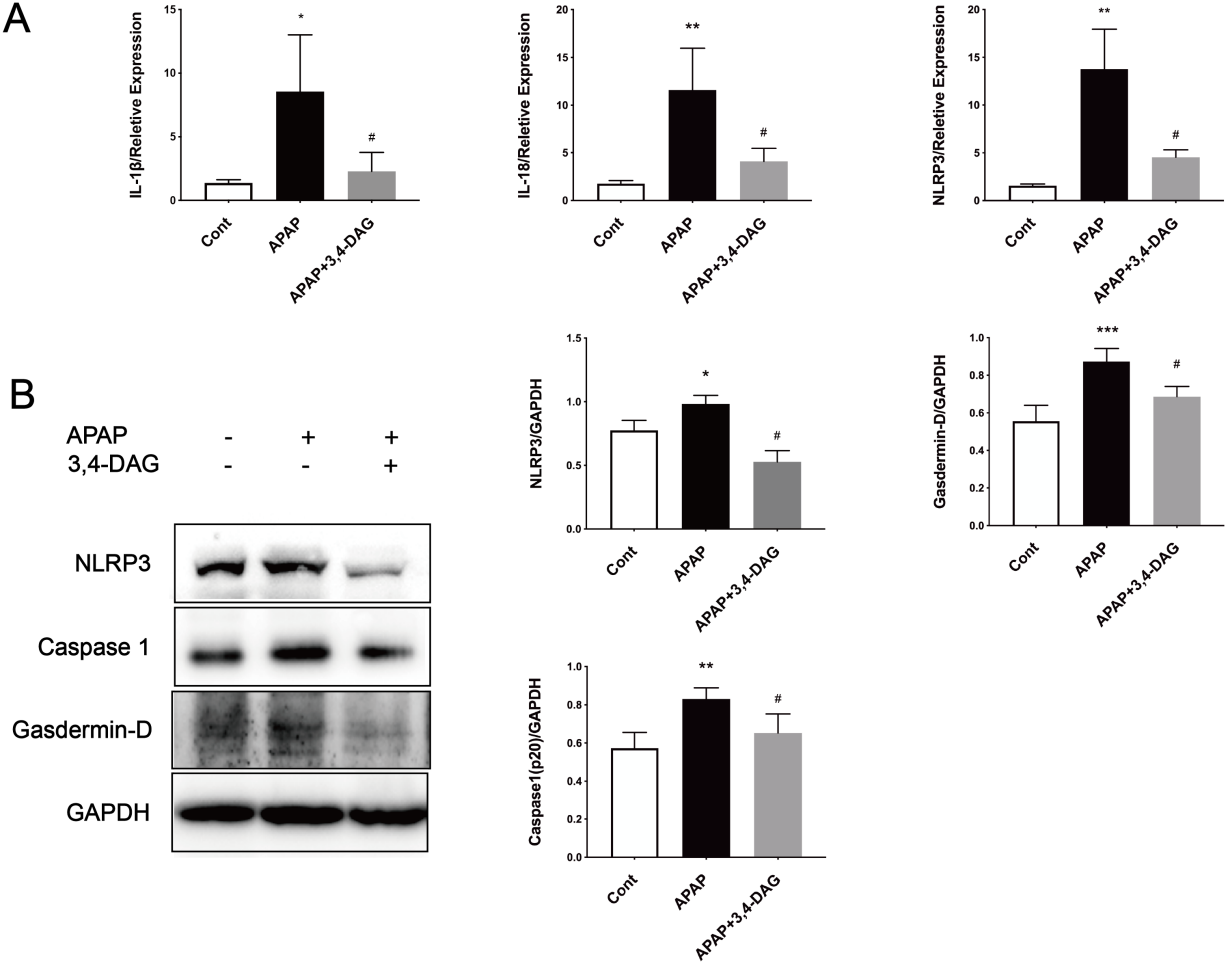
Effects of DAG on inflammatory factors and treatment on NLRP3, Gasdermin-D, Caspase1 (p20) activation in the liver tissues were determined 24 h after APAP-treated mice. (A) The expression of IL-1β, IL-18 and NLRP3 in the liver tissues. **p = 0.0022 APAP compared with control, ^#^*p* = 0.0054 compared with DAG (IL-1β). **p = 0.0005 APAP compared with control, ^#^*p* < 0.0001 (IL-18). **p = 0.0022 APAP compared with control, ^#^*p* = 0.0088 compared with DAG (*n* = 4 per group *df* = 3). (B) Proteins extracted from the liver were identified using western blot. DAG decreased the levels of NLRP3, Gasdermin-D, Caspase1 (P20) (B). *p = 0.0117 APAP compared with control, ^#^*p* < 0.0001 compared with DAG (NLRP3). ***p = 0.0003 APAP compared with APAP., ^#^*p* = 0.0113 compared with DAG (Gasdermin-D). **p = 0.0043 APAP compared with control, ^#^*p* = 0.0337 compared with DAG (Caspase 1-p20) (*n* = 3 per group *df* = 2).

It has been reported that APAP can cause pyrolysis of liver cells (Iorga & Dara, 2019). In order to further explore the mechanism of DAG on APAP-induced ALF, we extracted liver tissue RNA, and used RT-PCR to detect the relative expression of IL-1β, IL-18, and NLRP3. As shown in Fig. 6, compared with the control group, the expression of IL-1β, IL-18, and NLRP3 was significantly increased after APAP treatment. (*p* < 0.05). In contrast, DAG significantly inhibited the expression of IL-1β, IL-18, and NLRP3 (Fig. 6A) in the liver tissue of APAP-treated mice (*p* < 0.01). Moreover, compared with the control group, the expression of NLRP3, GSDMD, and Caspase1 (p20) in mouse liver tissue were upregulated after injection of APAP. In contrast, 100 mg/kg/day of DAG downregulated the expression of the above proteins in the liver tissue of APAP-treated mice (*p* < 0.01) (Fig. 6B). Therefore, we conclude that DAG can alleviate hepatocyte pyroptosis by inhibiting NLRP3/Caspase1 (p20)/GSDMD classical pyroptosis signaling pathway.

### DAG allayed the APAP-induced cytotoxicity in murine hepatocyte AML12 cells

**Figure 7:**
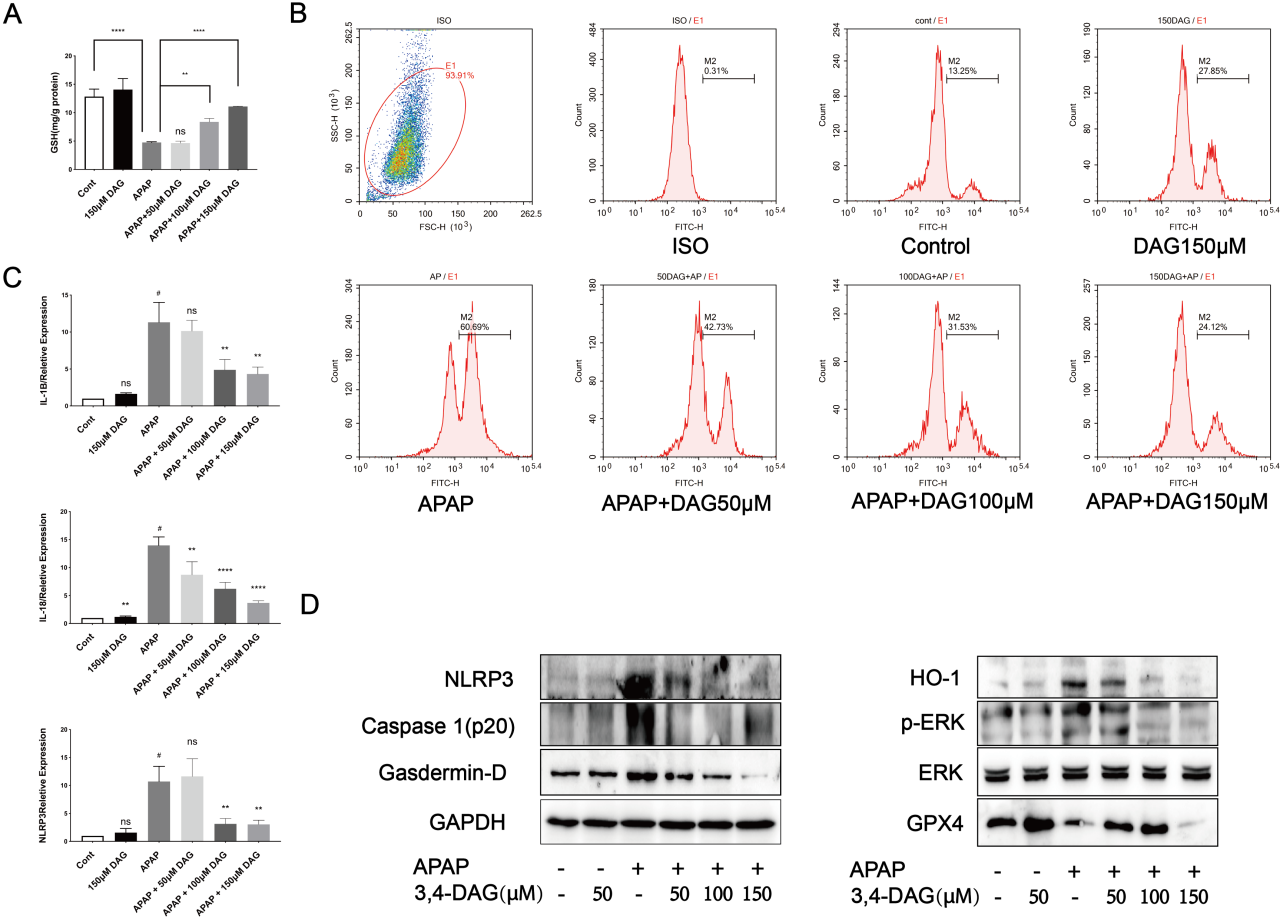
Effect of DAG on APAP-induced cytotoxicity in murine hepatocyte AML12 cells. (A) GSH content in AML12 cells. (****p < 0.0001 control compared with APAP and APAP compared with adding 150 µM DAG. **p < 0.05 APAP compared with adding 100 µM DAG) (*n* = 3 per group *df* = 2). (B) ROS in AML12 cells. (C) The expression of IL-1β, IL-18 and NLRP3 in AML12 cells. ^#^*p* < 0.05 APAP compared with control, **p < 0.005 APAP compared with adding 100 µM DAG, **p < 0.005 APAP compared with adding 150 µM DAG (IL-1β). ^#^*p* < 0.005 APAP compared with control, **p < 0.05 APAP compared with adding 50 µM DAG, ****p < 0.0001 APAP compared with adding 100 µM DAG, ****p < 0.0001 APAP compared with adding 150 µM DAG (IL-18). ^#^*p* < 0.005 APAP compared with control, **p < 0.005 APAP compared with adding 100 µM DAG, **p < 0.005 APAP compared with adding 150 µM DAG (NLRP3) (*n* = 3 per group *df* = 2). (D) Proteins extracted from AML12 cells were identified using western blot. DAG decreased the levels of p-ERK, HO-1, NLRP3, GSDMD and Caspase1 (p20), while at the same time it increased the levels of GPX4.

In order to further explore the effect of DAG and its concentration on liver cytotoxicity, AML12 cells were exposed to APAP (10 µM), DAG (50 µM, 100 µM, 150 µM). As shown in Fig. 7, compared with the control group, there was no significant change in GSH in the DAG group alone. The GSH level in the APAP group was significantly reduced (*p* < 0.001), and after adding DAG (100 µM, 150 µM), GSH increased to different degrees (*p* < 0.05). However, after adding 50 µM DAG, the GSH level did not change significantly. After that, flow cytometry was used to measure the content of ROS in each group of AML12 cells. Different concentrations of DAG reduced APAP-induced ROS production in AML12 cells to varying degrees, while DAG alone did not change significantly compared with the control group (Fig. 7, *p* < 0.05). Finally, After AML12 cells protein was extracted, NLRP3, GSDMD, Caspase1 (p20), ERK, P-ERK, GPX4 and HO-1 were determined by western blot. We confirmed that DAG could indeed alleviate the process of pyroptosis and ferroptosis of AML12 cells caused by APAP.

## Discussion

APAP overdose is one of the common causes of acute liver failure and has become a significant clinical problem in many Western countries  (Stravitz & Lee, 2019). Here, we investigated the effects of DAG on ALF induced by APAP in mice, and explored the possible underlying mechanisms. We found that APAP triggered changes in the histopathological structure of the liver, such as hepatocyte necrosis, loss of hepatocyte structure around blood vessels, and lymphocyte infiltration. However, the liver injury induced by APAP was improved by pretreatment with DAG. In addition, pretreatment with DAG significantly inhibited the increase in the AST and ALT enzyme level caused by APAP. We further found that DAG may protect against APAP-induced liver injury by suppressing ferroptosis and pyrolysis.

The protective effect of plant extracts on the liver is generally considered to be its antioxidant activity and the ability to improve the endogenous antioxidant defense system (Jaeschke, Xie & McGill, 2014). Our results show that DAG treatment increases the activity of SOD, CAT and the content of GSH in the liver of mice. It can be concluded that DAG can exert its antioxidant effect to reduce acute liver injury caused by APAP.

Recent studies have shown that ferroptosis plays a vital role in the pathogenesis of acute liver failure (Yamada et al., 2020). Lipid peroxidation (LPO) is frequently used as ferroptosis mechanism during APAP hepatotoxicity (Yoshioka et al., 2017). Studies have reported that the mitogen-activated protein kinase (MAPK) signaling pathway was associated with ROS-induced ferroptosis (Chang et al., 2021). Moreover, the expression of HO-1 related to ferroptosis was initially identified as cell death, and directly or indirectly inhibits glutathione peroxides 4 that occurs in cells by decreasing GSH levels (GPX4) (Kagan et al., 2017; Li et al., 2017). The acid-containing phospholipids of polyunsaturated lipopantothenic acid undergo a series of changes to produce lipid peroxides. Inhibition of GPX4 leads to the accumulation of iron-derived lipid peroxides, which leads to cell/subcellular membrane damage and ultimately cell death. Our results show that DAG significantly reduces the MDA level of APAP-induced ALF. In addition, we used western blot to detect the expression of p-ERK, HO-1, and GPX4 proteins in mouse liver tissues and AML12 cells protein. We found that DAG treatment upregulated the expression of GPX4 in APAP-induced ALF, and downregulated p-ERK and HO-1 expression. Therefore, we speculate that DAG can alleviate ALF by reducing ferroptosis caused by APAP.

Pyroptosis is an inflammatory form of programmed necrosis (Bergsbaken, Fink & Cookson, 2009; Jorgensen, Rayamajhi & Miao, 2017). The mechanism of APAP-induced pyrolysis of hepatocytes is not fully understood. It has demonstrated that damage associated molecular patterns (DAMPs) are released from liver cells, including mtDNA, nuclear DNA fragments, and ATP during APAP-induced-ALF (Woolbright & Jaeschke, 2017). The classical pathway of the pyroptosis includes recruiting and activating caspase-1, caspase-1 cuts and activates IL-18, IL-1β and other inflammatory factors, cutting the N-terminal sequence of gasdermin-D(GSDMD), and makes it bind to the membrane to produce a membrane pore, causing the pyroptosis (He, Hara & Núñez, 2016). Our results show that DAG treatment reduces the liver neutrophil infiltration and the expression of IL-1β, IL-18 and NLRP3. In addition, WB results showed that DAG treatment downregulated the levels of NLRP3, Caspase1 (p20), and GSDMD. These results indicate that DAG may also reduce the pyroptosis caused by APAP. Finally, GSH, PCR, WB, and flow cytometry were used to detect the GHS content, the expression of pyroptosis-related inflammatory factors, signaling pathways and ROS.

In conclusion, this study proved for the first time that DAG could effectively reduce liver damage induced by APAP in mice. Our data show that DAG effectively suppressed the levels of ALT and AST, histopathological changes and liver neutrophil infiltration and enhanced the activity of liver antioxidant enzymes (such as SOD, CAT and GSH). More interestingly, we also found that DAG can effectively resist APAP-induced hepatocyte ferroptosis, which may contribute to its protective effect on acute liver failure caused by APAP. These results show that DAG has great potential for development as a liver protective agent.

## Supplemental Information

10.7717/peerj.13082/supp-1 Supplemental Information 1 ARRIVE 2.0 checklist

10.7717/peerj.13082/supp-2 Supplemental Information 2 Raw Data of Liver weight ratio

10.7717/peerj.13082/supp-3 Supplemental Information 3 Raw Data of ALT

10.7717/peerj.13082/supp-4 Supplemental Information 4 Raw Data of AST

10.7717/peerj.13082/supp-5 Supplemental Information 5 Raw data of flow cytometry(C1) The file can be opened by FLOWjo software

10.7717/peerj.13082/supp-6 Supplemental Information 6 Raw data of flow cytometry(C2) The file can be opened by FLOWjo software

10.7717/peerj.13082/supp-7 Supplemental Information 7 Raw data of flow cytometry(C3) The file can be opened by FLOWjo software

10.7717/peerj.13082/supp-8 Supplemental Information 8 Raw data of flow cytometry(APAP1) The file can be opened by FLOWjo software

10.7717/peerj.13082/supp-9 Supplemental Information 9 Raw data of flow cytometry(APAP2) The file can be opened by FLOWjo software

10.7717/peerj.13082/supp-10 Supplemental Information 10 Raw data of flow cytometry(APAP3) The file can be opened by FLOWjo software

10.7717/peerj.13082/supp-11 Supplemental Information 11 Raw data of flow cytometry(APAP+3, 4-DAG1) The file can be opened by FLOWjo software

10.7717/peerj.13082/supp-12 Supplemental Information 12 Raw data of flow cytometry(APAP+3, 4-DAG2) The file can be opened by FLOWjo software

10.7717/peerj.13082/supp-13 Supplemental Information 13 Raw data of flow cytometry(APAP+3, 4-DAG3) The file can be opened by FLOWjo software

10.7717/peerj.13082/supp-14 Supplemental Information 14 Raw Data of Caspase 1(p20)

10.7717/peerj.13082/supp-15 Supplemental Information 15 Raw Data of ERK

10.7717/peerj.13082/supp-16 Supplemental Information 16 Raw data of GAPDH

10.7717/peerj.13082/supp-17 Supplemental Information 17 Raw Data of OH-1

10.7717/peerj.13082/supp-18 Supplemental Information 18 Raw Data of GSDMD

10.7717/peerj.13082/supp-19 Supplemental Information 19 Raw Data of GPX4

10.7717/peerj.13082/supp-20 Supplemental Information 20 Raw Data of p-ERK

10.7717/peerj.13082/supp-21 Supplemental Information 21 Raw Data of NLRP3

10.7717/peerj.13082/supp-22 Supplemental Information 22 Raw Data of CAT

10.7717/peerj.13082/supp-23 Supplemental Information 23 Raw Data of SOD

10.7717/peerj.13082/supp-24 Supplemental Information 24 Raw Data of GSH Mice

10.7717/peerj.13082/supp-25 Supplemental Information 25 Raw Data of GSH AML12

10.7717/peerj.13082/supp-26 Supplemental Information 26 Raw Data of IL-1B AML12

10.7717/peerj.13082/supp-27 Supplemental Information 27 Raw Data of IL-18 AML12

10.7717/peerj.13082/supp-28 Supplemental Information 28 Raw Data of NLRP3 AML12

10.7717/peerj.13082/supp-29 Supplemental Information 29 Raw Data of ROS iso

10.7717/peerj.13082/supp-30 Supplemental Information 30 Raw Data of ROS Control

10.7717/peerj.13082/supp-31 Supplemental Information 31 Raw Data of ROS 150uMDAG

10.7717/peerj.13082/supp-32 Supplemental Information 32 Raw Data of ROS APAP

10.7717/peerj.13082/supp-33 Supplemental Information 33 Raw Data of 50uMDAG+APAP

10.7717/peerj.13082/supp-34 Supplemental Information 34 Raw Data of APAP+100uMDAG

10.7717/peerj.13082/supp-35 Supplemental Information 35 Raw Data of ROS APAP+150uMDAG

10.7717/peerj.13082/supp-36 Supplemental Information 36 Raw Data of GAPDH

10.7717/peerj.13082/supp-37 Supplemental Information 37 Raw Data of NLRP3

10.7717/peerj.13082/supp-38 Supplemental Information 38 Raw Data of Caspase1(p20)

10.7717/peerj.13082/supp-39 Supplemental Information 39 Raw Data of GSDMD

10.7717/peerj.13082/supp-40 Supplemental Information 40 Raw Data of ERK

10.7717/peerj.13082/supp-41 Supplemental Information 41 Raw Data of p-ERK

10.7717/peerj.13082/supp-42 Supplemental Information 42 Raw Data of HO-1

10.7717/peerj.13082/supp-43 Supplemental Information 43 Raw Data of GPX4

## Additional Information and Declarations

### Data Availability

The following information was supplied regarding data availability: The original data and WB bands are available in the Supplementary Files. FCS files can be opened in FlowJo software https://www.flowjo.com/solutions/flowjo/.
