# Supplementary figures and images for: 3,4-dihydroxyphenylethyl alcohol glycoside reduces acetaminophen-induced acute liver failure in mice by inhibiting hepatocyte ferroptosis and pyroptosis
Source: PeerJ. 2022 Mar 14;10:e13082. doi: 10.7717/peerj.13082 (PMC8929172; doi:10.7717/peerj.13082)

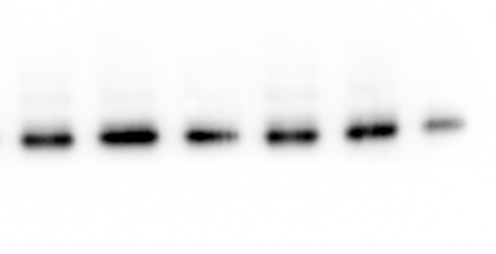

Supplement: Supplemental Information 14 [file peerj-10-13082-s014.tiff]

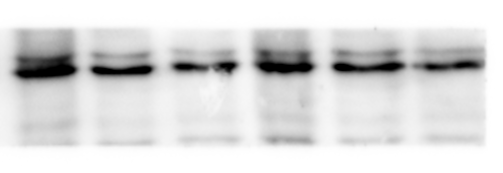

Supplement: Supplemental Information 15 [file peerj-10-13082-s015.tiff]

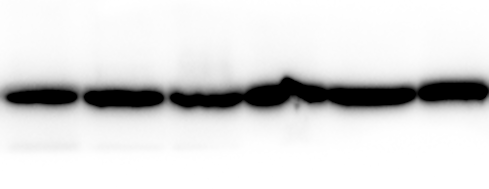

Supplement: Supplemental Information 16 [file peerj-10-13082-s016.tiff]

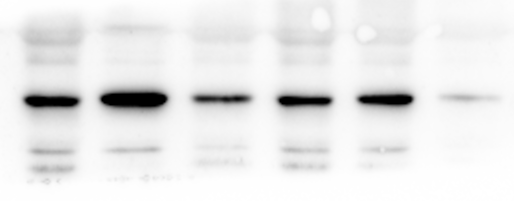

Supplement: Supplemental Information 17 [file peerj-10-13082-s017.tiff]

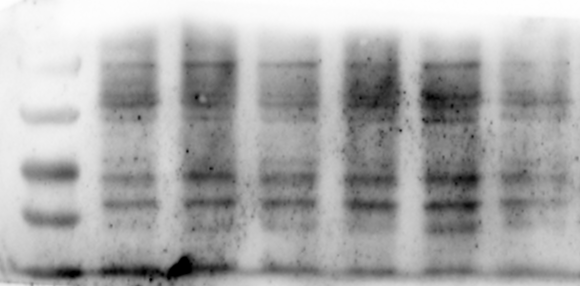

Supplement: Supplemental Information 18 [file peerj-10-13082-s018.tiff]

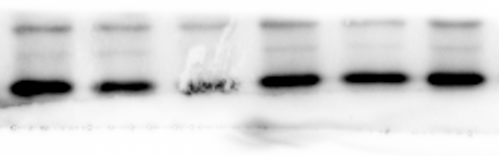

Supplement: Supplemental Information 19 [file peerj-10-13082-s019.tiff]

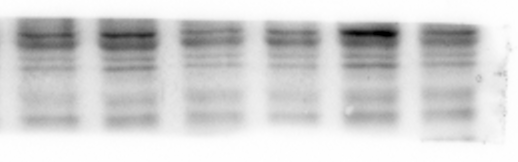

Supplement: Supplemental Information 20 [file peerj-10-13082-s020.tiff]

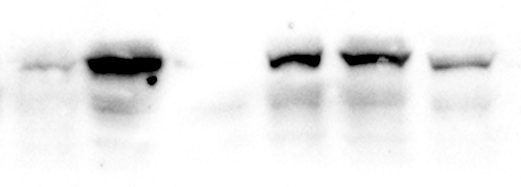

Supplement: Supplemental Information 21 [file peerj-10-13082-s021.tiff]

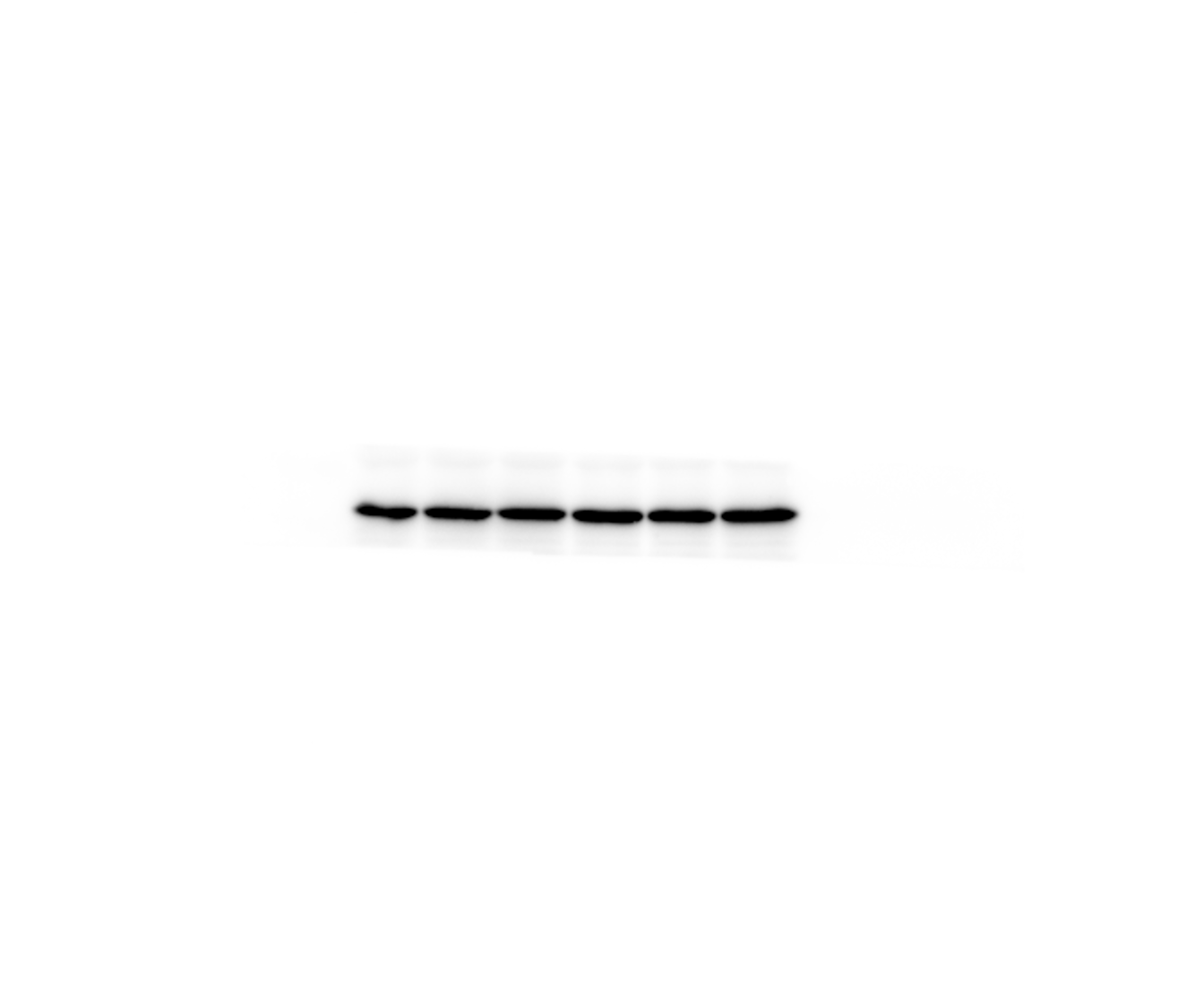

Supplement: Supplemental Information 36 [file peerj-10-13082-s036.tif]

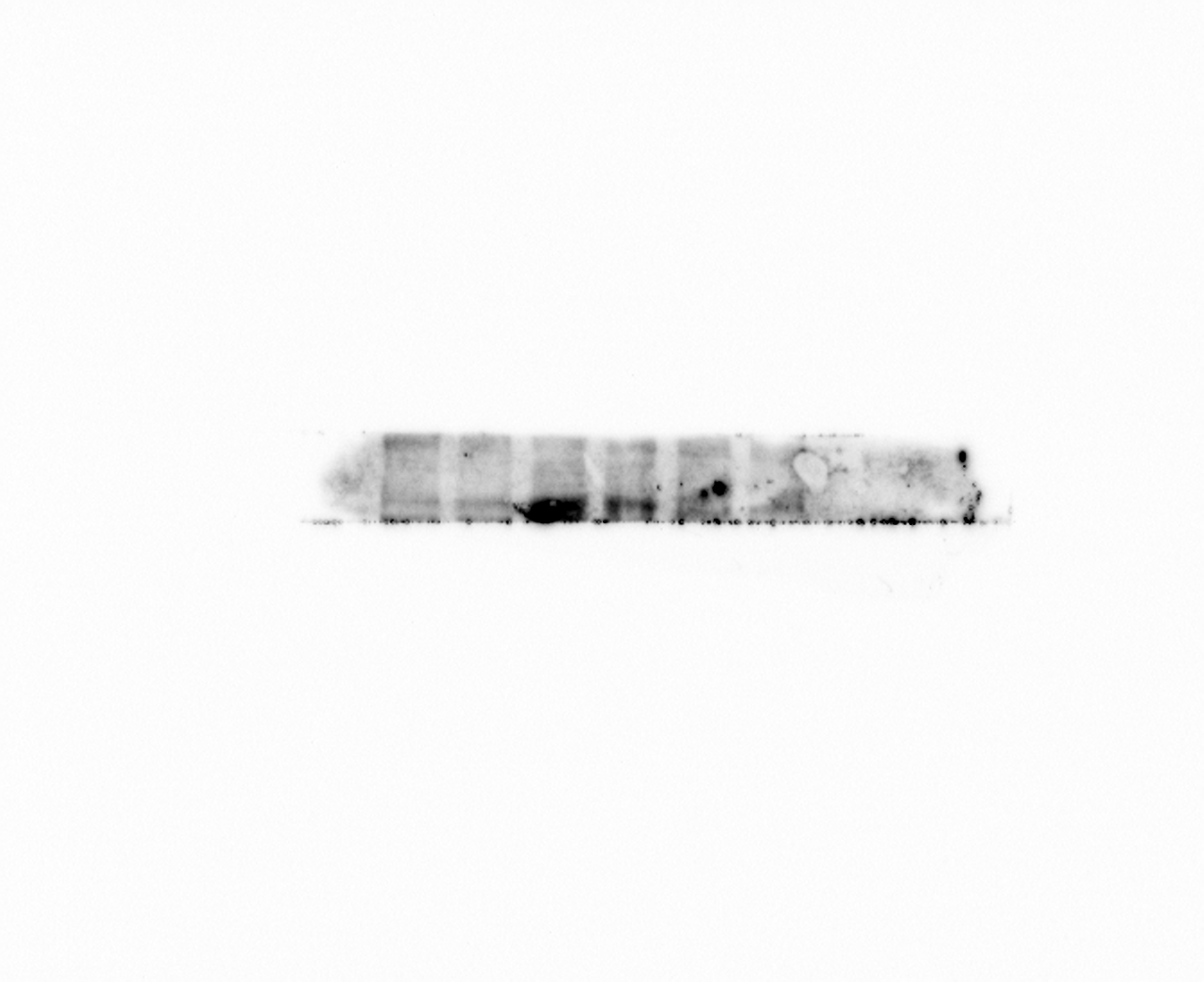

Supplement: Supplemental Information 37 [file peerj-10-13082-s037.tif]

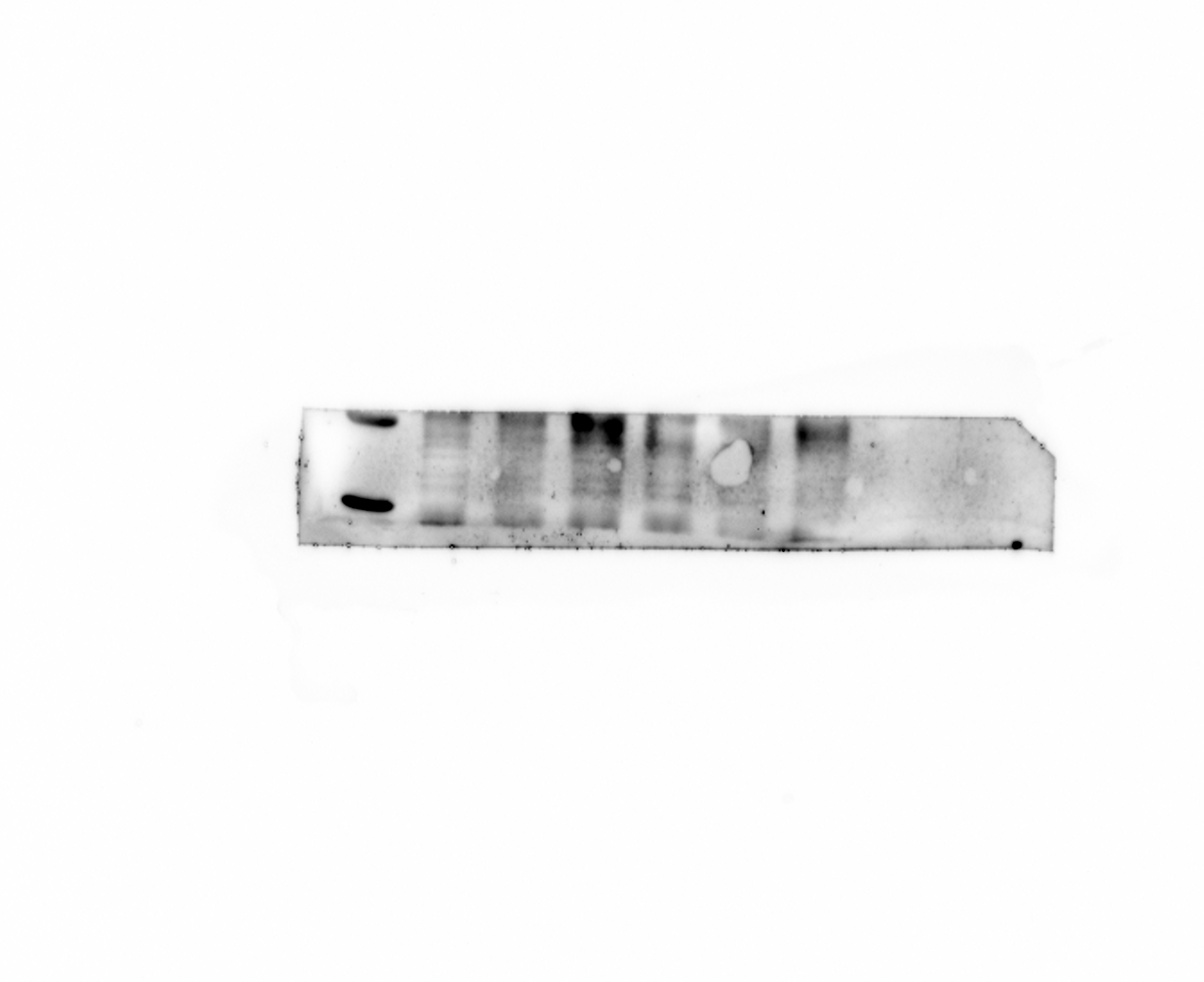

Supplement: Supplemental Information 38 [file peerj-10-13082-s038.tif]

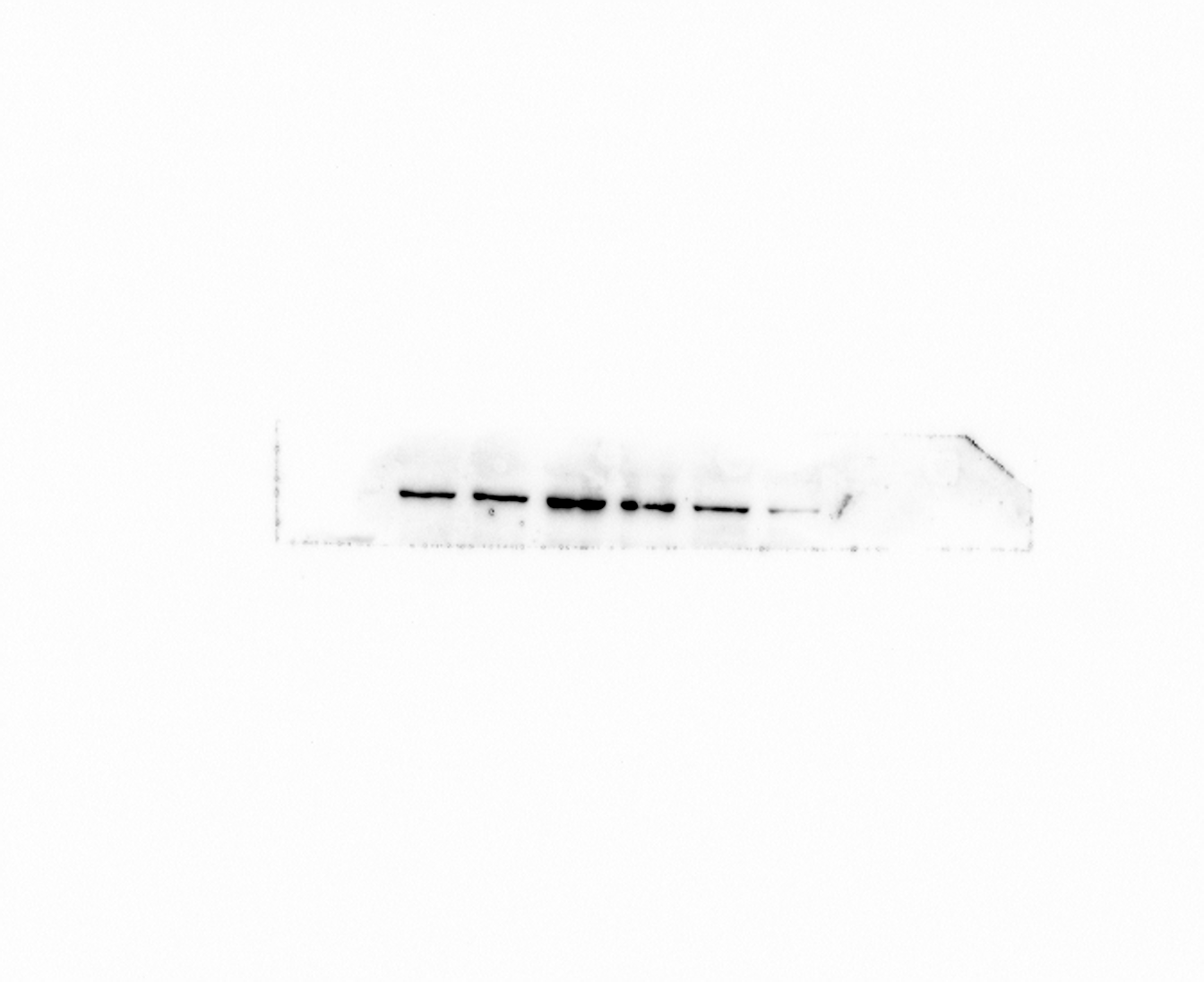

Supplement: Supplemental Information 39 [file peerj-10-13082-s039.tif]

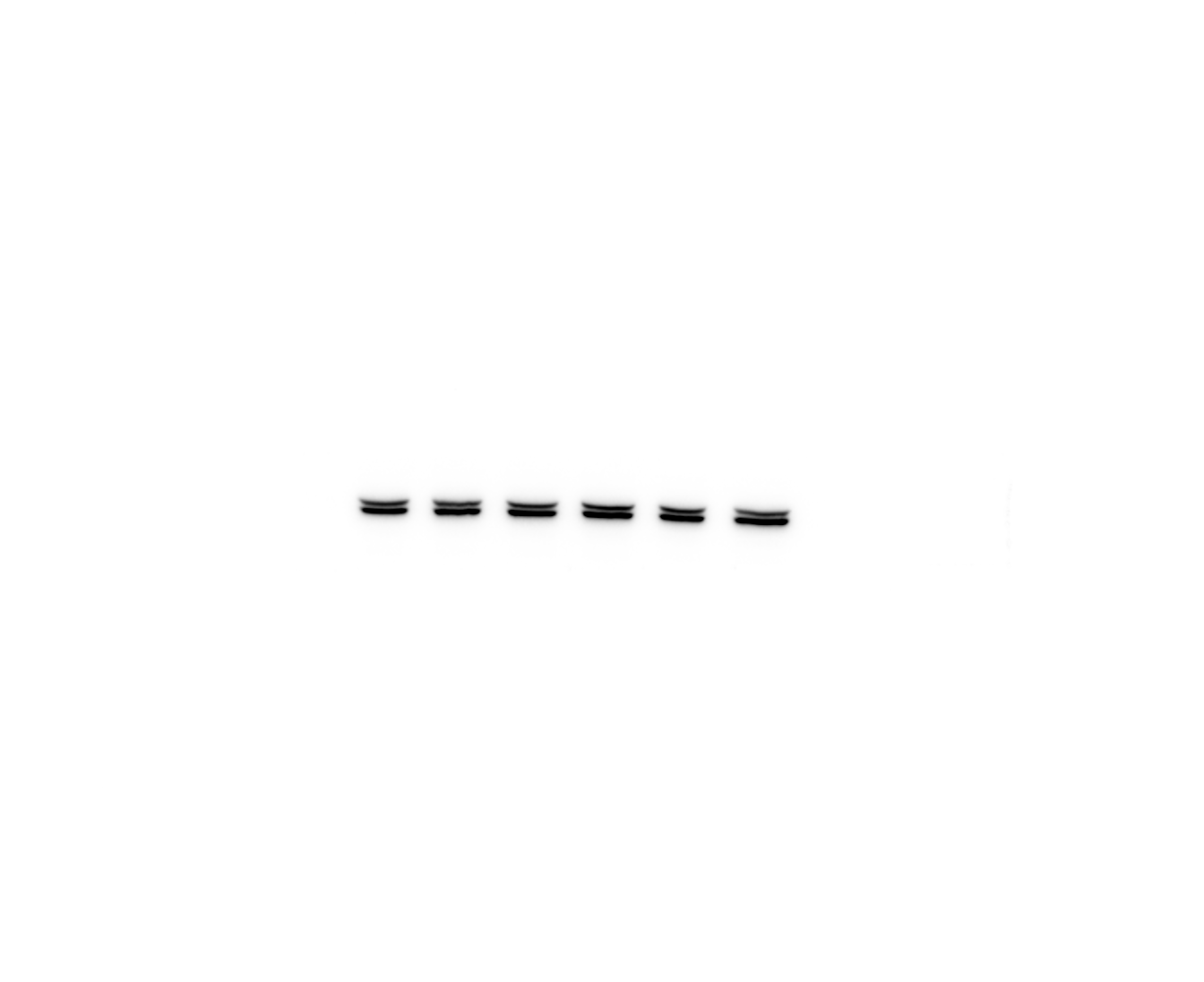

Supplement: Supplemental Information 40 [file peerj-10-13082-s040.tif]

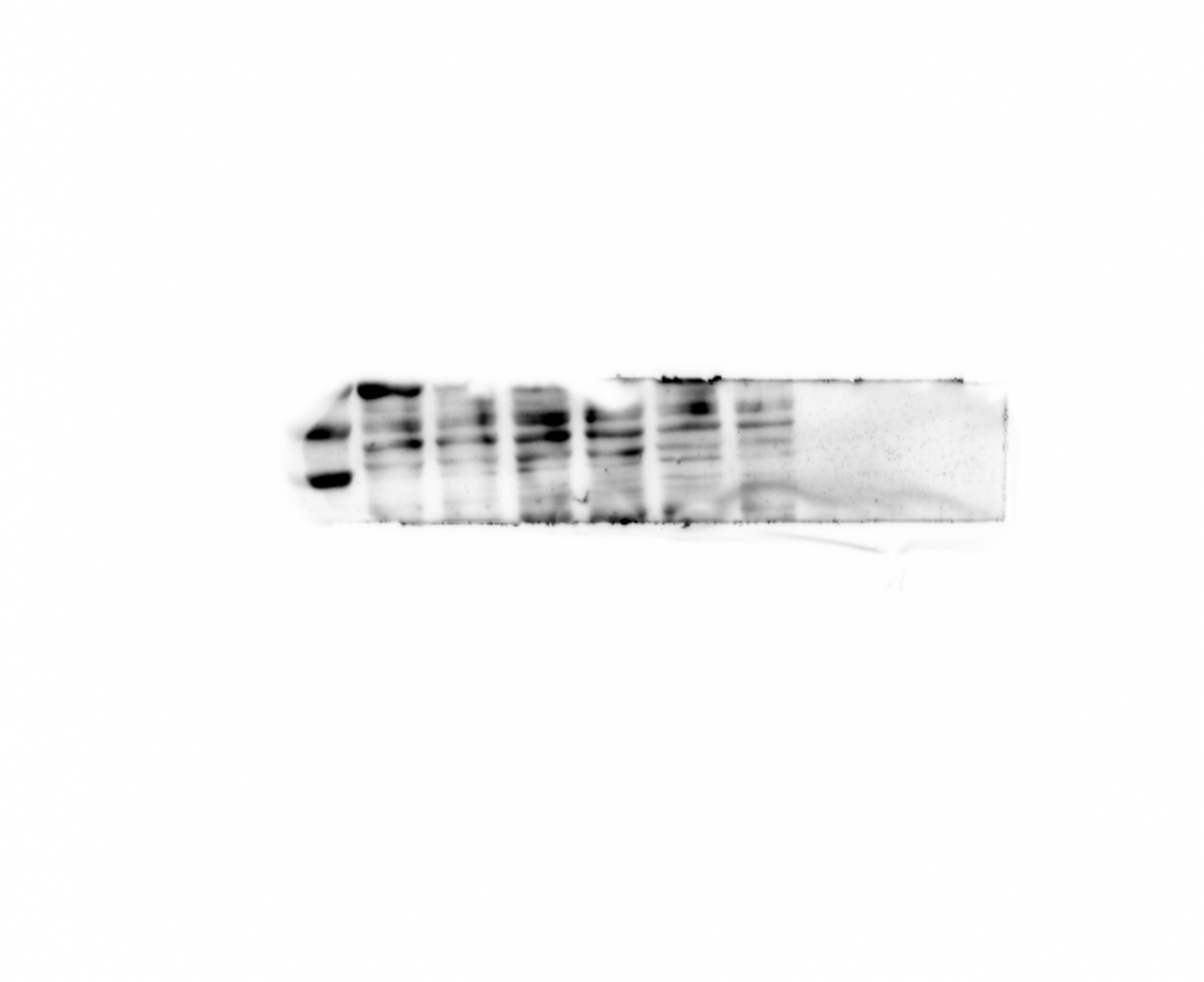

Supplement: Supplemental Information 41 [file peerj-10-13082-s041.tif]

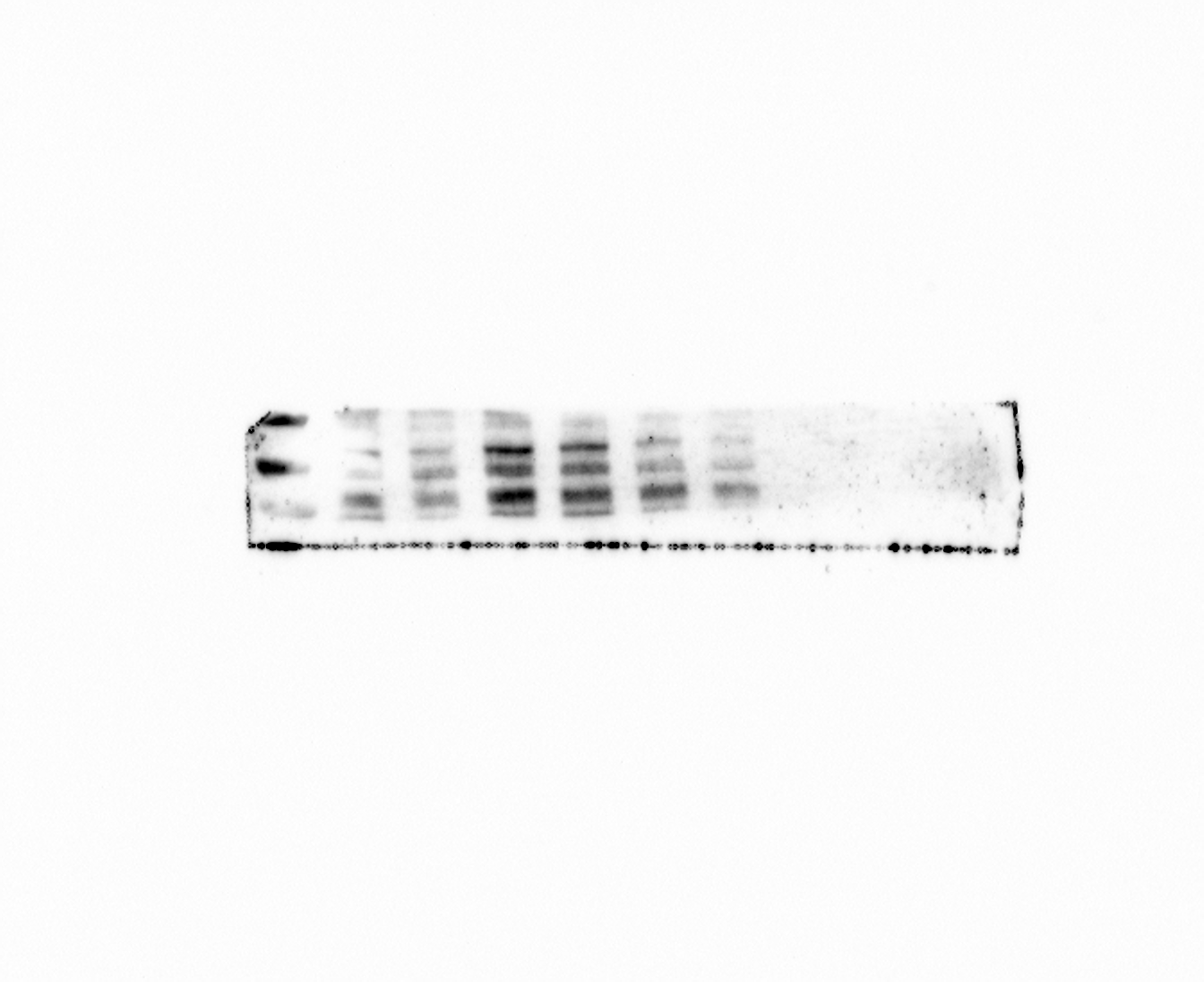

Supplement: Supplemental Information 42 [file peerj-10-13082-s042.tif]

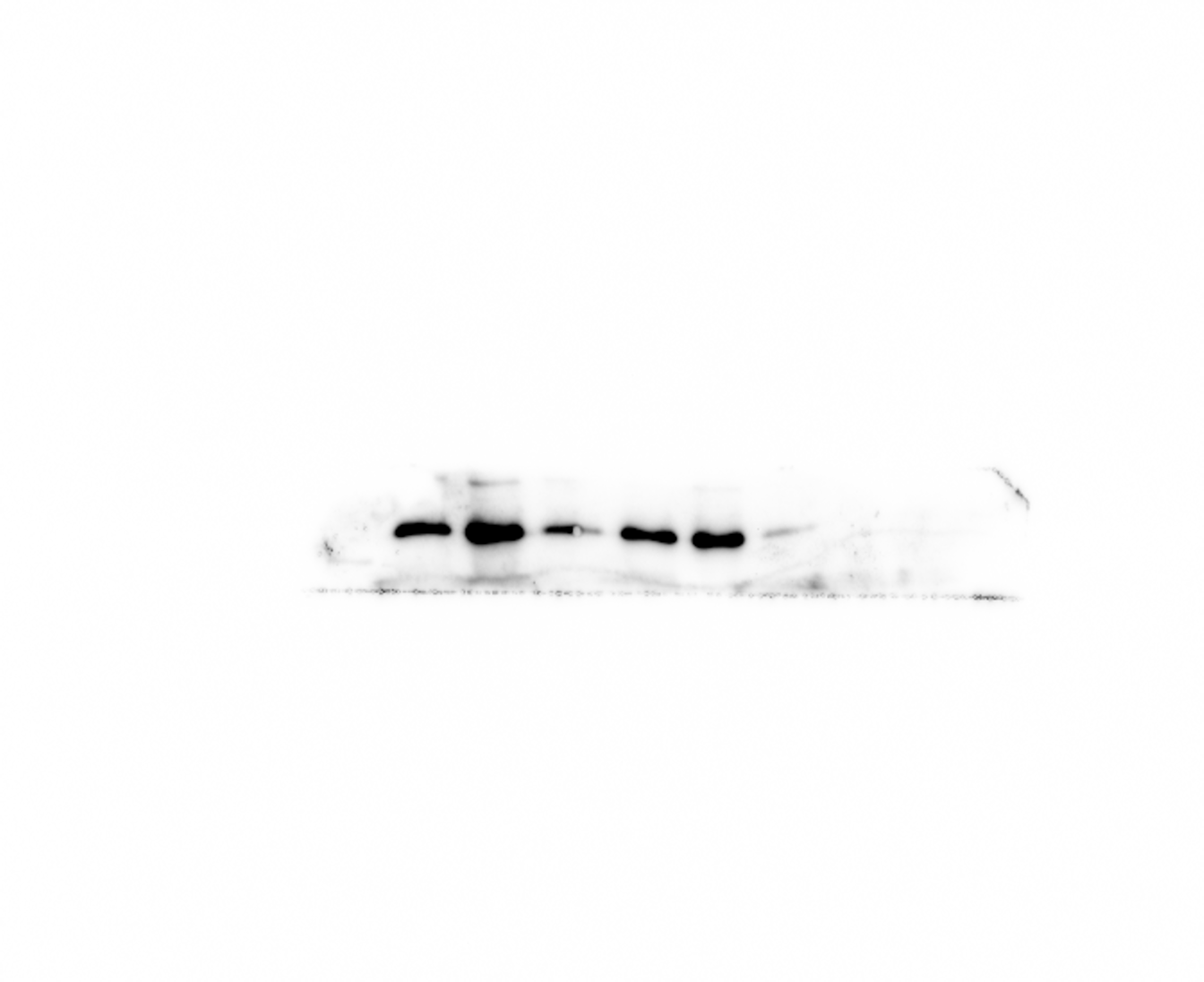

Supplement: Supplemental Information 43 [file peerj-10-13082-s043.tif]
